# A Hierarchical VLM-to-TD3 Framework with Novel Object Coordinate Estimation and Persistent Spatial Memory for Semantically Guided Indoor Navigation

**DOI:** 10.3390/s26154661

**Published:** 2026-07-23

**Authors:** Yernar Akhmetbek, Ayaulym Parmash, Temirlan Meiramkhanov, Azamat Yesmukhametov, Aigul Meirmanova, Darkhan Zholtayev

**Affiliations:** 1 Computer Science Department, SDU University, Almatinskaya St. 1/1, Almaty Region, Kaskelen 040900, Kazakhstan; yernar.akhmetbek@sdu.edu.kz (Y.A.); ayaulym.parmash@sdu.edu.kz (A.P.); 2 School of Artificial Intelligence and Data Science, Astana IT University, Mangilik Yel Ave., Astana 020000, Kazakhstan; 242813@astanait.edu.kz (T.M.); a.meirmanova@astanait.edu.kz (A.M.); 3 Institute of Smart Systems and Artificial Intelligence (ISSAI), Nazarbayev University, Qabanbay Batyr Ave., Block 1, Astana 010000, Kazakhstan; azamat.yeshmukhametov@nu.edu.kz

**Keywords:** vision-based navigation, deep reinforcement learning, vision–language models, autonomous mobile robot navigation

## Abstract

Autonomous semantic indoor navigation requires robust low-level control and high-level understanding of objects and spatial context in cluttered and partially occluded environments. While deep reinforcement learning (DRL) methods such as twin delayed deep deterministic policy gradient (TD3) enable reactive obstacle avoidance, they typically struggle with long-horizon semantic navigation, where object-location memory and language-level reasoning are required. We present a lightweight hierarchical two-stage framework that, to the best of our knowledge, is introduced for the first time to integrate a locally deployed vision–language model (VLM), semantic object coordinate memory, and a TD3-based DRL controller for language-conditioned indoor navigation. In Stage 1, the robot performs semantic exploration using odometry, 2D LiDAR, and VLM-based object recognition to build a geometric map and store detected object categories with their estimated world coordinates in a structured javaScript object notation (JSON) semantic memory. In Stage 2, a natural language query is used to retrieve the target object coordinates from memory and pass them to a TD3 target point navigation policy, which performs mapless navigation using odometry and RealSense RGB-D perception. The proposed framework combines open-vocabulary VLM-based object coordinate estimation, LiDAR mapping, RGB-D perception, and language grounding within a unified semantic memory representation. Experiments in a ROS-integrated realistic simulation demonstrate consistent goal-reaching performance and improved navigation efficiency compared with an Artificial Potential Field baseline using the same VLM and a DRL + GPT-4o mini configuration. We also compare the proposed VLM-based recognition module with YOLO-World v2.6 and Grounding DINO, showing that the VLM-based approach provides more reliable semantic grounding and target-coordinate estimation in the tested indoor navigation scenarios, particularly for flexible natural language object queries.

## 1. Introduction

### 1.1. Background and Motivation

Autonomous navigation for mobile robots is commonly characterized as an integrated capability to (i) perceive/map the environment, (ii) localize the robot within that environment, and (iii) plan and execute collision-free motion toward goals, often under dynamic and uncertain conditions [1,2,3]. Contemporary surveys emphasize that this functionality is realized through tightly coupled modules including sensor suites, sensor fusion, Simultaneous Localization and Mapping (SLAM), obstacle avoidance, path planning/following, and simulation/testing tools [3], while practical systems may explicitly decompose navigation into route planning, multisensor fusion for positioning/mapping, and automatic stopping/collision avoidance [1]. For many deployments, particularly indoors and in human-populated settings, navigation must additionally satisfy constraints beyond geometric feasibility, for example, human-aware or socially acceptable behaviors, thereby extending classical navigation objectives [4,5].

### 1.2. Deep Reinforcement Learning (DRL) and Visual Navigation

Navigation in unknown or dynamic environments can be framed as sequential decision-making where a policy maps onboard observations to motion commands while optimizing goal reaching, safety, and efficiency [6]. DRL leverages deep function approximation to learn such policies directly from high-dimensional observations and complex interaction dynamics, making it attractive for mapless navigation where explicit mapping and hand-engineered cost functions are difficult to maintain [7]. In practice, value-based DRL (e.g., deep Q-network (DQN)-style) has demonstrated target reaching with obstacle avoidance in simulation, while exploration-oriented DRL improves behavior in initially unknown spaces via learned state representations [6,7]. For wheeled robots requiring continuous control, actor–critic formulations are commonly adopted to better match nonholonomic kinematics and continuous-action interfaces, and they are sometimes paired with analytical controllers (e.g., Lyapunov-based tracking laws) to improve stability and tracking under navigation demands [8,9].

Visual DRL extends these ideas by using RGB/RGB-D observations to reduce sensor costs while increasing semantic and geometric information density for local decision-making [10]. Target-driven visual navigation often embeds the current observation and goal image into a shared space (e.g., Siamese actor–critic) to reach a visual goal without explicit map construction [11]. Sample efficiency can be improved by combining imitation learning with DRL (DIRL), and recent approaches have enhanced continuous-control DRL (e.g., twin delayed deep deterministic policy gradient (TD3)) with attention mechanisms for RGB-D target-driven navigation in crowded, dynamic settings [12,13]. However, many visual DRL systems remain effectively closed-set: generalization is constrained by the training distributions of targets/objects and visual appearances [11,13]. Accordingly, realistic deployment commonly relies on sim-to-real pipelines and robustness mechanisms that exploit simulation for scalable training and then transfer to physical robots [14,15], with extensions to multi-robot coordination and collision avoidance, and socially aware navigation among pedestrians when operating in human environments [16,17].

### 1.3. Foundation-Model-Enhanced Mobile Robot Navigation

Recent zero-shot object-goal navigation research has largely converged on pipelines that tightly couple online perception, mapping, and semantic reasoning throughout the execution. Most state-of-the-art systems assume continuous access to egocentric RGB-D sensing and maintain explicit spatial representations such as semantic occupancy grids, frontier maps, graphs, or top-view abstractions-that are repeatedly queried to select navigation subgoals [18,19,20,21,22]. While these structured approaches improve interpretability and exploration efficiency, they remain sensitive to perception noise, SLAM drift, and segmentation errors, and often require frequent large language model (LLM) or vision–language model (VLM) calls during navigation, which introduces latency, cost, and nondeterminism [21,22,23,24]. A number of methods emphasize semantic structure as the primary driver of exploration. Training-free semantic frontier pipelines combine classical mapping with language priors to guide search without policy learning, demonstrating that strong object-goal navigation (ObjectNav) performance is achievable without end-to-end reinforcement learning [18]. Vision–language frontier maps directly project VLM similarity scores onto spatial frontiers, enabling zero-shot semantic navigation; however, they still rely on persistent mapping and depth sensing at runtime [20,21]. Graph-based and region-based planners, including Voronoi abstractions and online 3D scene graphs, further structure the decision space but increase the system complexity and compound errors across perception, mapping, and reasoning modules [20,21]. Recent advances that exploit multimodal large language models to reason directly over top-view maps retain a tight coupling between perception, spatial memory, and decision-making [22].

Parallel to these structured approaches, predictive methods introduce imagination or world models to anticipate unseen space and reduce exploration inefficiency [25,26]. Although predictive reasoning can improve long-horizon planning, it amplifies concerns regarding model bias, sim-to-real transfer, and computational overhead, particularly when combined with foundation-model inference during navigation [6,11,24,26]. Multi-floor and hierarchical methods address vertical structures and large-scale environments but further increase the dependence on accurate global state estimation and repeated high-level reasoning [24,27].

In contrast, purely reactive systems take no direction from mapping; rather, they interpret the visual–language information and translate it directly into short-term motion directives. Open-vocabulary navigation based solely on reflexes has shown that the ability to interpret the location of objects using multidirectional eyesight and the ability to use previously trained VLMs make it possible to follow a command without a map or a learned model. However, this type of method cannot find its way in uneven terrains or cluttered environments, and they do not exhibit consistent long-range movement because the systems do not possess the capability to maintain the memory of their locations over time [28]. Frameworks based on helpers are specifically designed to compensate for these problems, which have been addressed through a system of supplementary aids for obstacle avoidance, navigational discovery, and target detection, but still rely on constant vigilance of the environment through direct imaging as well as a non-synchronous interaction between modules of functionality [29].

In contrast to both continuously mapped pipelines and fully reactive systems, our approach adopts a two-stage hybrid paradigm that explicitly decouples semantic perception from the navigation execution. In the first stage, the robot uses LiDAR and RGB sensing to construct a 2D metric map of the environment, detect and localize objects, and store object names with their estimated coordinates derived from the VLM. This stage leverages reliable classical robotics tools—standard robot operating system (ROS) mapping and control stacks—to ensure geometric consistency and accurate spatial anchoring, while confining VLM usage to a bounded exploration phase. Unlike prior work that repeatedly queries foundation models during navigation [21,22,23], semantic reasoning is amortized into a compact object-level memory.

In the second stage, the system deliberately abandons the map and raw sensor semantics, retaining only object identities and coordinates. An LLM operates solely over this symbolic spatial memory for target selection and task intent without access to visual inputs or global maps. Navigation to selected targets is then executed by a mapless deep reinforcement learning policy, trained specifically for the room-scale environment, which relies only on odometry-based forward pose estimation. We use the term “mapless” to describe the Stage-2 navigation policy, which does not access the occupancy grid or any metric map constructed in Stage 1. Instead, the TD3 agent receives only the goal coordinates retrieved from the symbolic object memory, together with real-time depth observations and odometry. Thus, although Stage 1 produces a map for exploration, the navigation controller itself operates without any map representation, relying entirely on reactive depth-based control and coordinate-level goal specification. This design contrasts with map-dependent planners [18,20,30] and predictive world-model approaches [25,26] by isolating learning-based control from perception and by avoiding continuous SLAM or semantic updates during execution.

By separating semantic understanding from motion control, the proposed pipeline addresses several recurring limitations identified in prior zero-shot navigation systems. First, it reduces the sensitivity to perception noise and SLAM drift during navigation by freezing spatial semantics after exploration [18,21]. Second, it eliminates runtime dependence on VLM/LLM inference for low-level decision-making, mitigating the latency and cost concerns highlighted in foundation-model-based planners [22,23,24]. Third, it enables robust long-horizon behavior through a trained DRL policy that operates maplessly using odometry, avoiding the brittleness of purely reactive VLM control [28] while remaining simpler than fully predictive world-model frameworks [25,26]. Finally, by operating on object-level symbolic memory rather than dense maps, the approach naturally supports open-vocabulary reasoning and instance-level generalization emphasized in recent semantic learning and augmentation studies [31,32,33,34], while remaining compatible with hierarchical and multi-floor extensions [24,27].

Taken together, this hybrid mapping-to-mapless navigation paradigm situates our framework between structured semantic exploration and learned reactive control, offering a practical compromise that leverages foundation models where they are the most effective—semantic interpretation and reasoning—while entrusting execution to efficient, reliable, and deployment-ready control policies [35,36].

### 1.4. Motivation and Paper Contribution

Most of the reviewed studies that address semantic object navigation rely on SLAM during the navigation phase in conjunction with conventional local path planners. To achieve reliable navigation performance, many of these approaches employ multiple environment representations, such as 2D occupancy grids alongside 3D scene reconstructions or room-level maps, which significantly increases computational complexity and system overhead [18,19,21,25,30,35]. In addition, several studies have adopted multiple language models for different roles, including policy execution, high-level reasoning, and planning [22,23,26,27,29], or combined VLMs, such as contrastive language–image pre-training (CLIP) and bootstrapping language–image pre-training (BLIP) with LLMs for object recognition and semantic grounding [20,26,30,33]. A further limitation is that many approaches depend on large-scale LLMs or VLMs accessed via external APIs, often requiring continuous internet connectivity and remote servers during operation [20,26,35,36]. Other works increased architectural complexity by employing multiple visual encoder–decoder blocks to process perceptual inputs [32]. Considering the limitations of prior SLAM-heavy and multi-model architectures, our contribution introduces a simplified yet conceptually validated semantic navigation framework with clearly differentiated system-level design choices. The principal contributions of this study are as follows:

- **ROS-based semantic navigation framework in a more realistic simulation environment:** Unlike many existing LLM- and VLM-based semantic navigation studies evaluated mainly in abstracted simulators such as Gibson, Habitat, or HM3D, this work demonstrates a complete semantic navigation pipeline in a ROS/Gazebo-based robotic simulation environment, including robot kinematics, sensor topics, odometry, 2D LiDAR, RGB-D perception, and navigation execution.
- **Framework-level integration of semantic grounding, spatial memory, and DRL navigation:** The main novelty is not the introduction of a new VLM or DRL algorithm, but the design and evaluation of a hierarchical framework that connects VLM-based semantic interpretation and target localization, SLAM-assisted persistent semantic coordinate memory, and TD3-based goal-directed navigation using compact goal-relative observations.
- **Persistent semantic coordinate memory for object-level navigation:** During exploration, the robot combines 2D LiDAR-based mapping, odometry, RGB-D sensing, and VLM-based object recognition to estimate and store object categories with their world coordinates in a structured javaScript object notation (JSON) semantic memory, enabling target retrieval even when the object is no longer visible.
- **Decoupled semantic exploration and mapless execution:** The system separates semantic environment understanding from navigation control. In the first stage, the robot builds semantic memory using mapping and VLM-based perception. In the second stage, the TD3 policy performs target point navigation using onboard depth perception, odometry, and relative goal information without requiring a global planner during execution.
- **Edge-deployable VLM-based grounding and detector comparison:** This work evaluates semantic grounding alternatives, including GPT-4o mini and locally deployable Moondream. This analysis highlights the suitability of an edge-deployable VLM for natural language-driven semantic navigation in which flexible human commands must be converted into structured target information.
- **Baseline comparison and rigorous stress-test evaluation:** To isolate the contribution of the navigation component, the proposed Moondream + DRL framework was compared with a Moondream + artificial potential field (APF) baseline under the same VLM-based target localization module. In addition, the framework is evaluated through rigorous tests involving object-level stress scenarios, obstacle-rich navigation, and sign-based semantic navigation, including stop signs, toilet signs, and other sign-directed tasks. These evaluations assess not only navigation reliability but also the ability of the proposed framework to ground diverse semantic targets and execute goal-directed navigation in more challenging ROS-based environments.

### 1.5. Paper Structure

The remainder of the paper is organized as follows. Section 2 presents and describes the simulation environment used in this study. Section 3 details the exploration phase, during which the robot scans the environment to detect objects and estimate their semantic labels and spatial coordinates. Section 4 introduces the proposed object navigation framework, including the integration of the VLM with a TD3-based navigation agent. This section explains the TD3 architecture, training procedure, reward formulation, and action and observation spaces, as well as their interactions with the VLM module. Section 5 reports the experimental evaluation and testing phase, including generalization experiments across different room layouts and object configurations. Section 7 discusses the limitations of the proposed approach and outlines directions for future research. Finally, Section 8 concludes the paper.

## 2. Methodology and Simulation Environment

### 2.1. Methodology Overview for a Generic Mobile Robot

Indoor service robots—such as robotic vacuums and household assistants—are increasingly expected to follow natural language commands (e.g., “go to the chair” or “find the bottle”) and operate reliably in cluttered, previously unseen rooms. Achieving this capability requires more than geometric obstacles. Avoidance: the robot must ground semantic concepts in physical space and then execute safe and efficient motion toward the requested goal.

**Figure 1:**
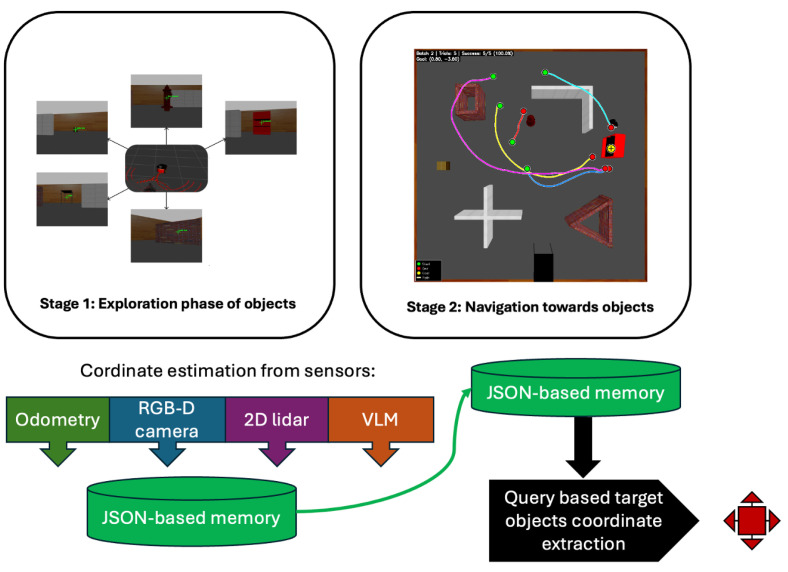
Two-stage workflow overview of the proposed semantic navigation framework: semantic exploration → goal-directed navigation.

As shown in Figure 1, to address this need, we employ a two-stage, decoupled pipeline that links language understanding with low-level control. In Stage 1 (Exploration and Semantic Memory), the robot explores while collecting RGB-D observations, (optionally) 2D LiDAR, and odometry/TF data. A prompted VLM performs open-vocabulary object recognition, and detected objects are converted into spatial coordinates using depth geometry and the robot pose estimate. The results are stored in a lightweight JSON semantic memory (e.g., object name, position, confidence, and timestamp). In Stage 2 (Query-Driven Navigation), an LLM retrieves the target object coordinates from the JSON memory and provides them to a mapless DRL controller, which uses local depth and proprioception to generate collision-aware velocity commands for goal reaching.

We evaluate the system in a ROS-integrated realistic simulation using repeated object-goal trials and report success rate (SR), success weighted by path length (SPL) (and SPL for successful trials only), finish time, and final localization error. Comparisons include a classical ROS baseline using APF control with the same VLM perception and a DRL+GPT-4o mini configuration. We further assess generalization by introducing unseen objects and recomputing all metrics under identical conditions.

### 2.2. Simulation Environment

**Figure 2:**
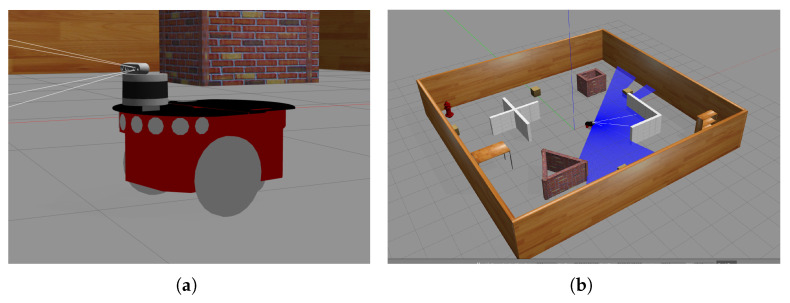
The simulation setup: (**a**) depicts the Pioneer 3-DX robotic platform equipped with the Hokuyo LiDAR and RealSense camera; (**b**) the integrated Gazebo simulation environment used to evaluate navigation among multiple semantic objects.

A high-fidelity simulation framework based on ROS Noetic running on Ubuntu 20.04 is used for the experimental validation. The Pioneer 3-DX (Figure 2a), a differential-drive mobile robot extensively used in autonomous navigation research, serves as the main robotic platform.

The robot is outfitted with a variety of sensors intended for comprehensive perception in order to facilitate strong spatial awareness and interaction. An Intel RealSense D435i camera provides high-resolution RGB-D data, and a Hokuyo 2D LiDAR is used for precise geometric obstacle detection. These sensors are crucial to the robot’s perception system because they give it the depth data required for object grounding and 3D mapping while also enabling it to discriminate between navigable space and intricate obstacles.

The onboard odometry system is an essential part of the navigation logic. The platform continuously estimates the robot’s instantaneous pose, including global coordinates (x,y) and orientation (θ), by monitoring wheel encoder data.

The TD3.world environment, an indoor arena created by Cimurs et al., serves as the simulation’s host [37] (displayed in Figure 2b). This setting offers a 5 m×5 m confined space intended to assess spatial reasoning and collision avoidance. The world is populated with a number of unique objects in order to assess the system’s semantic abilities: “Bookshelf”, “Fire Hydrant”, “Table”, and “Block”. RViz is used for debugging and real-time visualization. It shows the robot’s internal state, including processed point clouds, sensor transforms, and the active target point.

## 3. Exploration and Semantic Mapping Strategy (Stage 1)

Most of the reviewed works discussed in the Introduction that deploy VLMs or LLMs for navigation do not adopt a structured separation between semantic exploration and control. In contrast, our framework explicitly decouples the exploration and semantic mapping phase (Stage-1) from the navigation phase (Stage-2). Stage-1 is dedicated to systematic environment scanning and semantic memory construction, whereas Stage-2 performs goal-directed navigation using the stored symbolic coordinates.

Although conventional object detection networks such as BLIP, CLIP, or you only look once (YOLO)-based models could be employed for object coordinate estimation, our objective is not merely detection. The VLM in Stage-1 is intentionally utilized as an open-vocabulary, language-conditioned visual grounding module rather than a closed-set class predictor. This enables semantic alignment between natural language queries and visual observations without being restricted to a predefined label set. While a standard detector combined with odometry can localize known object categories in world coordinates, such approaches lack flexibility when navigation targets are expressed through dynamic or environment-specific language descriptions.

The exploration and semantic mapping pipeline operates in a sequential, integrated flow to bridge the gap between low-level navigation and high-level semantic understanding. Initially, the robot uses the Gmapping algorithm to perform SLAM, constructing a 2D occupancy grid while navigating through unknown space. During this process, color images acquired from the Intel RealSense D435i RGB-D sensor are uploaded to the VLM (GPT-4o mini or Moondream) for semantic analysis. It is worth noting that Stage-1 semantic mapping operates on the full-resolution RGB images (1024×768) from the RealSense sensor for VLM-based object grounding, whereas the single-channel depth tensor 64×64 is exclusively used as a compact state representation for the DRL navigation controller in Stage-2.

**Figure 3:**
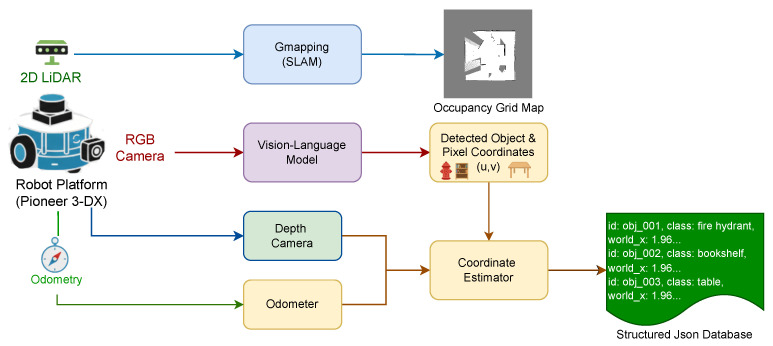
The proposed exploration and coordinate retrieval pipeline. The system integrates Gmapping for SLAM, VLM for semantic identification, and a coordinate estimator to fuse odometry and depth data into a persistent JSON database.

Using the VLM, target objects such as a bookshelf, hydrant, table, or block are identified (e.g., bookshelves, fire hydrants, tables, and blocks) and their corresponding pixel coordinates (u,v) in images are retrieved. The VLM then receives synchronized pixel values with up-to-date information from the odometry and depth camera, which are sent to the Coordinate Estimator module. The Coordinate Estimator converts the pixel coordinates into 3D global coordinates and saves them in a structured JSON format, which enables the system to create a permanent record of the spatial location of each identified object throughout time. The pipeline is illustrated in Figure 3.

**Figure 4:**
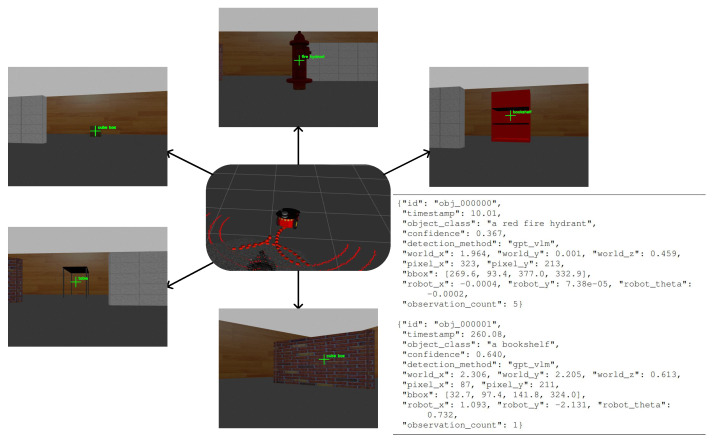
Pioneer 3-DX in simulation with active LiDAR scanning.

The VLM outputs 2D pixel coordinates (u,v) for the detected objects. These coordinates are then synchronized with real-time data from the odometry and depth camera. As shown in the integrated visualization in Figure 4, the system performs real-time detection across multiple viewpoints while maintaining a consistent global map. The coordinate estimator module subsequently converts these pixel values into 3D global coordinates, which are saved in a structured JSON format to create a permanent record of the environment’s semantic layout.

### 3.1. SLAM and Frontier Exploration

A real-time 2D occupancy grid map produced by Gmapping [38] serves as the foundation for the autonomous navigation framework. The robot finds “frontiers”—the lines separating known free space from uncharted territory—instead of using manual waypoints to cover the surroundings methodically. The system distinguishes between navigable free space, static obstacles, and unknown regions while maintaining accurate localization through the use of Gmapping.

### 3.2. Coordinate Estimation from VLM Output

The robot needs a 3D metric position in relation to the global map in order to carry out navigation tasks, even though the VLM supplies the semantic class and 2D pixel location. The VLM output (u,v) is transformed into global coordinates $P_{world}\in R^{3}$ by our transformation pipeline.

#### 3.2.1. Robust Depth Extraction

Statistical neighborhood sampling technique used to reduce sensor noise or invalid readings (NaNs) in the point cloud. We define a 5×5 pixel search window *W* for a detected object center (u,v). Let *S* represent the collection of legitimate 3D points that were extracted from this window’s point cloud:

$$S=\{P_{i}\in R^{3}|\left(u_{i},v_{i}\right)\in W,\mathrm{isValid}\left(P_{i}\right)\}$$

To reject outliers, the object’s position in the camera frame, $P_{cam}$, is computed as the dimension-wise median of *S*:

$$P_{cam}=\mathrm{median}\left(S\right)={\left[x_{c},y_{c},z_{c}\right]}^{T}$$

#### 3.2.2. Robust Depth Extraction via Non-Parametric Estimation

Commodity depth sensors, such as the Intel RealSense D435i, produce structured point clouds that are corrupted by two principal noise sources: (i) quantization and temporal jitter intrinsic to the active-IR stereo pipeline, and (ii) depth discontinuities at object boundaries, where neighboring pixels can return distances to surfaces that differ by more than a meter. Because these errors are non-Gaussian and heavy-tailed, a simple arithmetic mean over a pixel neighborhood is statistically inappropriate: even a single outlier can shift the estimate far from the true object surface.

We therefore adopt a dimension-wise median estimator, which belongs to the family of $L_{1}$-norm (least-absolute-deviation) estimators and possesses a breakdown point of 50%—meaning that up to half of the samples in the neighborhood can be arbitrarily corrupted without affecting the result [39]. For a detected object center (u,v), we define a 5×5 pixel search window *W* and extract the set *S* of valid 3D points from the registered point cloud:

(1) $$S=\left\{P_{i}={\left[x_{i},y_{i},z_{i}\right]}^{T}\in R^{3}\;|\;\left(u_{i},v_{i}\right)\in W,\;\mathrm{isValid}\left(P_{i}\right)\right\}$$

where isValid(·) rejects NaN entries and points at zero or maximum sensor range. The camera frame position estimate is then:

(2) $$P_{\mathrm{cam}}=\mathrm{med}\left(S\right)={\left[\mathrm{med}\left(\left\{x_{i}\right\}\right),\;\mathrm{med}\left(\left\{y_{i}\right\}\right),\;\mathrm{med}\left(\left\{z_{i}\right\}\right)\right]}^{T}$$

Window size. The 5×5 window (|W|=25 pixels) is chosen to balance two competing objectives. A larger window increases the number of valid depth samples, improving robustness to sensor dropout, but also increases the risk of including background surfaces that lie behind the target object. At a typical operating distance of 1–3m with the RealSense D435i (horizontal field of view $\approx 87^{\circ }$, image width 640px), a 5×5 patch subtends approximately 3–8cm on the object surface—small enough to remain within a single object for the target classes in our experiments (fire hydrant, bookshelf, table), yet large enough to yield a statistically meaningful sample even when 30–40% of pixels return invalid readings.

#### 3.2.3. Coordinate Frame Transformation via Rigid-Body Kinematics

The point $P_{\mathrm{cam}}$ is expressed in the optical frame convention adopted by ROS and the RealSense SDK, in which the *Z*-axis points forward (into the scene), the *X*-axis points rightward, and the *Y*-axis points downward. To fuse this measurement with the robot’s odometric pose, we must transform it into the world frame, which uses the East–North–Up (ENU) convention standard in mobile robotics. This transformation is decomposed into two successive rigid-body motions, each belonging to the Special Euclidean Group SE(3).

Step 1: Optical frame → Robot base frame.

The optical-to-base transformation accounts for two distinct geometric effects:

1. Basis change (rotation). The ROS robot base frame follows the convention *X*-forward, *Y*-left, *Z*-up, whereas the camera optical frame uses *Z*-forward, *X*-right, *Y*-down. The rotation that maps the optical axes to the base axes is derived by aligning each axis:
  - The camera’s *Z* (forward) must align with the base’s *X* (forward), giving the first column of the rotation matrix.
  - The camera’s *X* (right) must align with the base’s −Y (left), giving the second column.
  - The camera’s *Y* (down) must align with the base’s −Z (up), giving the third column.
  Writing the resulting rotation matrix $R_{\mathrm{opt}\to \mathrm{base}}$ column-by-column:
  (3) $$R_{\mathrm{opt}\to \mathrm{base}}=\left[\begin{array}{ccc} 0 & 0 & 1\\ -1 & 0 & 0\\ 0 & -1 & 0 \end{array}\right]$$
  This matrix is orthogonal with det(R)=+1, confirming that it is a proper rotation (an element of SO(3)) and preserves handedness. Its structure—composed entirely of {0,±1} entries—reflects the fact that the two frames differ by a $90^{\circ }$ axis permutation rather than an arbitrary rotation, which is a direct consequence of the standardized axis conventions in ROS.
2. Mounting offset (translation). The camera is physically displaced from the robot’s base-link origin by a translation vector $T_{\mathrm{offset}}\in R^{3}$, which encodes the camera’s mounting position along the base frame’s *X* (forward), *Y* (lateral), and *Z* (vertical) axes. This offset is measured from the robot’s URDF (Unified Robot Description Format) model or from physical calibration.

Combining both effects as an SE(3) transformation:

(4) $$P_{\mathrm{base}}=R_{\mathrm{opt}\to \mathrm{base}}\;\cdot \;P_{\mathrm{cam}}+T_{\mathrm{offset}}$$

- Step 2: Robot base frame → World frame.

The robot’s instantaneous pose in the world frame is provided by the odometry system as a position $\left(x_{r},y_{r}\right)$ and a yaw angle ψ (rotation about the vertical *Z*-axis). For a differential-drive ground robot operating on a planar surface, roll and pitch are negligible, and the full SE(3) transformation reduces to an SE(2) planar rigid-body motion embedded in $R^{3}$:

(5) $$P_{\mathrm{world}}=R_{z}\left(\psi \right)\;\cdot \;P_{\mathrm{base}}+t_{r}$$

where the rotation matrix $R_{z}\left(\psi \right)\in \mathrm{SO}\left(3\right)$ and translation vector $t_{r}$ are:

(6) $$R_{z}\left(\psi \right)=\left[\begin{array}{ccc} \cos\psi & -\sin\psi & 0\\ \sin\psi & \cos\psi & 0\\ 0 & 0 & 1 \end{array}\right],\;t_{r}=\left[\begin{array}{c} x_{r}\\ y_{r}\\ 0 \end{array}\right]$$

The matrix $R_{z}\left(\psi \right)$ is the standard generator of planar rotations: it preserves the *Z*-component (height) while rotating the horizontal (X,Y) components by the yaw angle ψ. This follows from the fact that the Pioneer 3-DX operates under a planar motion constraint—its wheels maintain contact with a flat floor—so the only degree of rotational freedom affecting the horizontal projection of detected objects is the heading angle.

Complete transformation chain.

Composing both steps, the full mapping from pixel-level VLM output to world coordinates is:

(7) $$P_{\mathrm{world}}=R_{z}\left(\psi \right)\left[R_{\mathrm{opt}\to \mathrm{base}}\;\cdot \;\mathrm{med}\left(S\right)+T_{\mathrm{offset}}\right]+t_{r}$$

This expression is a composition of two elements of SE(3), which is itself an element of SE(3), guaranteeing that distances and angles are preserved throughout the chain (isometric mapping). The decomposition into two explicit stages—rather than a single pre-composed transformation—is deliberate: it allows the camera offset $T_{\mathrm{offset}}$ to be calibrated independently of the odometry, and it permits each stage to be validated separately during system integration.

#### 3.2.4. Error Sources and Sensitivity

The accuracy of $P_{\mathrm{world}}$ is governed by three independent error sources, whose contributions compound through the transformation chain:

1. VLM pixel error $\delta_{uv}$: Displacement of the reported pixel center from the true object center. At a depth *z*, a pixel error of $\delta_{u}$ pixels translates to a lateral world error of approximately $\delta_{u}\;\cdot \;z/f_{x}$, where $f_{x}$ is the camera’s horizontal focal length in pixels.
2. Depth sensor noise $\delta_{z}$: The RealSense D435i depth error grows quadratically with range ($\sigma_{z}\propto z^{2}$). The median estimator suppresses outliers but does not eliminate systematic bias.
3. Odometric drift $\delta_{\psi },\delta_{xy}$: Accumulated heading error $\delta_{\psi }$ introduces a lateral displacement that grows linearly with the object distance $∥P_{\mathrm{base}}∥$, i.e., $\delta_{\mathrm{world}}\approx ∥P_{\mathrm{base}}∥\;\cdot \;\delta_{\psi }$.

The temporal filtering mechanism described in Section 3.3 mitigates these errors by averaging over multiple independent observations acquired from different robot poses, thereby reducing the variance of the final estimate at a rate governed by the EMA smoothing factor.

### 3.3. Ground Truth and Estimated Coordinate Acquisition

To evaluate localization accuracy, both ground truth and VLM-estimated object coordinates are collected independently. Ground truth coordinates are obtained directly from the Gazebo simulator by querying the world state via the /gazebo/get_model_state service, which returns the precise 3D pose $\left(x_{gt},y_{gt},z_{gt}\right)$ of each object model in the world frame. These coordinates are deterministic and unaffected by sensor noise, serving as the reference baseline for localization error computation.

Estimated coordinates are produced by the VLM-based coordinate estimation pipeline described in Section 3. During the exploration phase, each time the stop-and-sense mechanism is triggered, the VLM identifies objects in the captured RGB image and returns their pixel-space centers (u,v). These 2D pixel coordinates are then fused with the synchronized depth point cloud and the robot’s odometric pose through the coordinate frame transformation chain (Equations (1)–(3)) to yield estimated world coordinates $\left(x_{est},y_{est},z_{est}\right)$, which are stored in the JSONL database. The localization error $E_{loc}$ is computed as the Euclidean distance between the ground truth and estimated positions:

(8) $$E_{loc}=\sqrt{{\left(x_{gt}-x_{est}\right)}^{2}+{\left(y_{gt}-y_{est}\right)}^{2}}$$

This metric is reported in the 2D plane (ignoring the *z* component), as the navigation goal coordinates are specified as $\left(x_{g},y_{g}\right)$.

### 3.4. Prompted VLM-Based Open-Vocabulary Object Coordinate Estimation

The detection method begins during the stop-and-sense phase, which was discussed in Section 3. An RGB image created from the camera of a robot is transformed into a Base64 string format and sent to either the GPT-4o mini or Moondream backbones to compare their performance.

A constructed prompt is required to make sure that the VLM performs spatially grounded object localization instead of simply providing a general description of the image. To do this, the prompt restricts what the model can produce to a specific list of objects (e.g., “fire hydrant”, “brick box”, and “blue box”) and also provides a defined format for the response. An example of the prompt template developed for this study is provided in Listing 1:

**Listing 1.** System Prompt used for VLM Object Detection.

You are a precise object localization system. Analyze this {width}x{height} image.


TASK: Identify standalone, movable objects from the list:

 - fire hydrant

 - brick box

 - blue box


INSTRUCTIONS:

-  You may only report objects using exact names from the list above.

-  If an object is present but not listed, ignore it.

-  If unsure about mapping, skip instead of guessing.


For EACH detected object, return:

1. Exact model name (must match list spelling character-for-character)

2. Pixel X center of the object (0 = left, {width} = right)

3. Pixel Y center of the object (0 = top, {height} = bottom)


FORMAT (one per line): object_name:[x,y]


Now analyze the image:



This grounded feedback allows the system to pause navigation or trigger a re-exploration if the VLM determines the target is no longer at the estimated coordinate or is obscured.

### 3.5. Data Persistence and Object Mapping

Every detection that has been verified becomes part of a continuously maintained database by the robot as it conducts exploration. The information is transformed using a particular standardized text format, JSON Lines (.jsonl). This transformation of data makes the information utilizable during subsequent processing, such as for the creation of maps using geometrically referenced points around detected objects, and for searching for data based on different characteristics.

Each entry reflects a separate instance of an identified physical object (the description of which changes whenever additional observations are made).

To describe how these sets of data are stored together, in the Database Schema shown in Table 1, we can see that they generate the full picture of the location and type of detected objects using data from both VLMs and navigation systems (ie., Spatio-Semantic Schemata).

The Listing 2 shows an example of two different occurrences when fire hydrants were detected previously in our system. As you can see, obj_000001 has been seen different times and as such has been more accurately located than obj_000000, which is a new observation.

**Listing 2.** Sample JSONL Output showing two detected objects.

{"id": "obj_000000",

 "timestamp": 10.01,

 "object_class": "a red fire hydrant",

 "confidence": 0.367,

 "detection_method": "gpt_vlm",

 "world_x": 1.964, "world_y": 0.001, "world_z": 0.459,

 "pixel_x": 323, "pixel_y": 213,

 "bbox": [269.6, 93.4, 377.0, 332.9],

 "robot_x": -0.0004, "robot_y": 7.38e-05, "robot_theta": -0.0002,

 "observation_count": 5}


{"id": "obj_000001",

 "timestamp": 260.08,

 "object_class": "a bookshelf",

 "confidence": 0.640,

 "detection_method": "gpt_vlm",

 "world_x": 2.306, "world_y": 2.205, "world_z": 0.613,

 "pixel_x": 87, "pixel_y": 211,

 "bbox": [32.7, 97.4, 141.8, 324.0],

 "robot_x": 1.093, "robot_y": -2.131, "robot_theta": 0.732,

 "observation_count": 1}



### 3.6. VLM-Driven Data Acquisition

To incorporate semantic understanding into the exploration process while maintaining high operational efficiency, we utilize an Asynchronous Snapshot Acquisition pipeline. Data is captured atomically in a synchronized buffer, including RGB visual data, 3D point cloud data, and the robot’s current odometry pose, allowing for semantic mapping without interrupting the robot’s continuous motion.

The acquisition cycle functions as follows:

1. Interval Trigger: The robot operates on a fixed time interval of t=1 s. This high-frequency trigger ensures dense semantic coverage of the environment during transit.
2. Asynchronous Snapshotting: Once the timer expires, the system captures a synchronized “snapshot” of all sensor streams. Unlike discrete “stop-and-sense” methods, the robot does not cease its navigation activities; instead, the frozen data is offloaded to a parallel processing thread.
3. Parallel Visual Processing: The captured snapshot is sent to the VLM for analysis. Benchmarking results (across 100 requests) indicate an average inference latency of 0.4397s (max: 0.54s). The VLM identifies target classes and returns 2D pixel coordinates, which are mapped to the synchronized 3D point cloud to determine global world coordinates.
4. Continuous Motion: Because the detection and coordinate estimation occur in a background process, the robot maintains its exploration trajectory without latency-induced pauses.

## 4. Semantically Guided Object Navigation Using DRL and VLM, (Stage 2)

We created our framework using the TD3 algorithm as part of our effort to develop a robust local navigation solution for dynamic environments via DRL. Our work builds on similar architectures that have been developed by others, focusing on goal-driven exploration [37]; however, we have made considerable changes in the input data types; instead of simply using sparse LiDAR observations, we now incorporate a much larger and more complex input space that includes not just vision depth sensing but also proprioception.

### 4.1. Action and Observation Spaces

The DRL agent interacts with the environment through a high-dimensional observation space and a continuous action space, enabling precise control of the Pioneer 3-DX platform.

#### 4.1.1. Observation Space

The observation vector $s_{t}$ is multimodal, combining visual perception with proprioceptive and geometric data to provide the agent with a comprehensive understanding of its surroundings. The input consists of:

- Visual Input: A 64×64 RGB image captured by the RealSense camera, providing rich semantic and spatial features of the environment.
- Scalar Input: A 7-dimensional array of scalars representing the robot’s state and its relationship to the goal:
  $$S=[v_{prev},\omega_{prev},v_{last},\omega_{last},d_{goal},\theta_{goal},l_{min}]$$
  where $v_{prev}$ and $\omega_{prev}$ are the previous linear and angular velocities, $v_{last}$ and $\omega_{last}$ are the most recent commands, $d_{goal}$ is the Euclidean distance to the target, $\theta_{goal}$ is the relative heading angle to the target, and $l_{min}$ is the minimum distance reading from the realsense depth camera d435 sensor for immediate obstacle detection.

#### 4.1.2. Action Space

To operate within the framework of an ongoing action space, $a_{t}=\left[v,\omega \right]$, where *v* denotes linear speed constrained to the range [0,1] and omega signifies rotation speed normalized to the range [−1,1], the following strategy was implemented. Linear speed must remain stable in its continued motion towards the front, but may also assist in achieving stability while learning; thus, it had to be improved to ensure a stable forward velocity in the motion of robots in order to achieve maximum efficiency. Rotation speed indicates a directional change to the end.

The mapping of these continuous variables from *v* and ω directly to the motor controller of the robot enables the ability to provide a symmetrical and simultaneous path signal for a smooth and fluid trajectory during navigation.

### 4.2. Reward Function Design

The reward function is the critical component governing the agent’s behavior. We designed a composite function $R_{total}$ that balances goal-seeking behavior with safety constraints:

(9) $$R_{total}=R_{event}+R_{motion}+R_{safety}$$

Here, $R_{\mathrm{event}}$ is the terminal reward the robot receives when a collision occurs or when the goal is reached. Sparse rewards are assigned for trial termination outcomes:

(10) $$R_{event}=\left\{\begin{array}{cc} +100 & \mathrm{if}\;\mathrm{Goal}\;\mathrm{Reached}\\ -100 & \mathrm{if}\;\mathrm{Collision}\;\mathrm{or}\;\mathrm{Stuck}\\ 0 & \mathrm{otherwise} \end{array}\right.$$

Here, $R_{\mathrm{motion}}$ denotes the motion-efficiency reward that encourages smooth trajectories; the agent is rewarded for high linear velocities ($v_{t}$) and penalized for excessive rotational velocities ($\omega_{t}$):

(11) $$R_{motion}=\frac{v_{t}}{2}-\frac{|\omega_{t}|}{2}$$

The next one is the dynamic safety penalty ($R_{safety}$) reward. It ensures safe navigation in confined spaces. We implemented a hybrid wall-avoidance penalty that accounts for both absolute proximity and the rate of approach. Let $d_{t}$ be the distance to the nearest obstacle and $d_{safe}=1.0\;m$. Let $\Delta d=d_{t}-d_{t-1}$ represent the change in distance between consecutive timesteps.    

(12) $$R_{safety}=P_{prox}+P_{approach}$$

where the static proximity penalty $P_{prox}$ is:

(13) $$P_{prox}=\left\{\begin{array}{cc} -\frac{d_{safe}-d_{t}}{2} & \mathrm{if}\;d_{t}<d_{safe}\\ 0 & \mathrm{otherwise} \end{array}\right.$$

and the dynamic approach penalty $P_{approach}$ punishes the agent only if it is actively moving closer to an obstacle (Δd<0):

(14) $$P_{approach}=-2.0\;\cdot \;\max\left(0,-\Delta d\right)$$

This formulation allows the robot to operate near walls when necessary (e.g., passing through doors) but penalizes actions that drive it strictly closer to a collision.

### 4.3. Actor-Critic Network Architecture

Mobile robotic systems that typically utilize the standard DRL methods make use of Multi-Layer Perceptrons (MLPs)s to interpret low-dimensional LiDAR data. In order to extract all of the spatial information from the RealSense camera, a new Dual Stream Feature Extractor architecture was developed. This provides a method to extract visual and numerical data simultaneously, then combine them to make a single decision.

**Figure 5:**
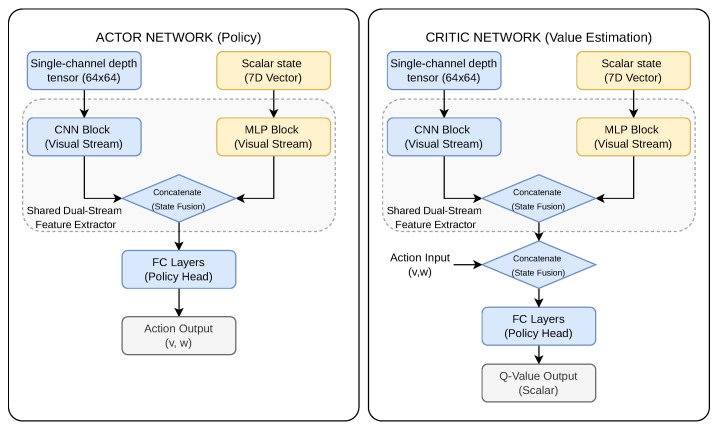
TD3 Model: actor and critic network structure.

The architecture is shown in Figure 5 and it consists of two first-level streams:

1. Visual Stream: The input is a single-channel depth tensor image resized to 1×64×64 pixels. This stream utilizes a Convolutional Neural Network (CNN) similar to the Mnih-DQN architecture [40]:
  - Conv1: 32 filters, 8×8 kernel, stride 4, ReLU.
  - Conv2: 64 filters, 4×4 kernel, stride 2, ReLU.
  - Conv3: 64 filters, 3×3 kernel, stride 1, ReLU.
  The output is flattened into a 64-dimensional feature vector representing the local obstacle geometry.
2. Scalar Stream (MLP): A 7-dimensional vector containing robot kinematics (linear/angular velocities) and relative goal coordinates is processed through a two-layer MLP (7→64→64) with ReLU activations.

The outputs from both streams are concatenated into a single 128-dimensional latent vector, which is then passed through a fully connected fusion layer (128→256) to inform the actor and critic networks.

### 4.4. DRL Training Parameters

The navigation policy was trained in the TD3.world Gazebo environment using the TD3 algorithm. In contrast to traditional solutions that make use of a series of sparse (1D) LiDAR scans, our agent was trained to produce continuous velocity commands from images representing depths with dimensions of (64×64) pixels.

The training occurred over 200,000 time steps using the Stable-Baselines3 framework. High exploration rates within a generalized high-dimensional state were achieved by setting both high-action noise to 0.8 and a larger-than-average batch size of 256 for training. Additional hyperparameter information may be found in Table 2.

**Figure 6:**
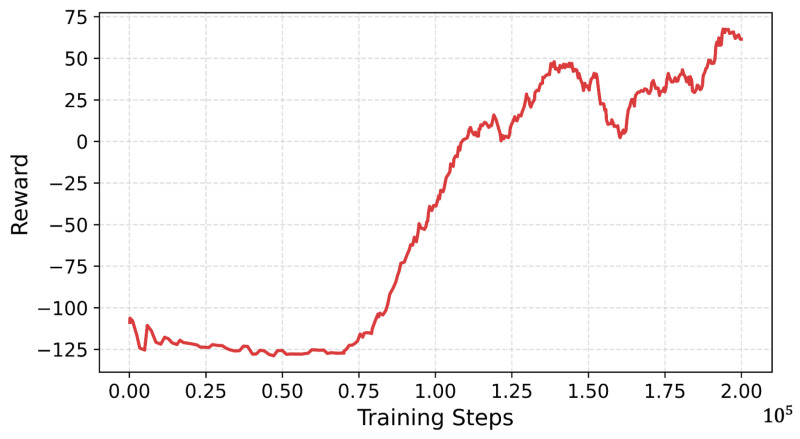
Training Cumulative Reward.

Figure 6 shows the development of an agent using TD3 through a plot of its cumulative reward curve, which depicts the learning process as moving through a series of rewards (lower, up, and down, due to random exploration) to become goal-directed behavior of reaching a set goal or objective. As training continued after 200,000 time steps, the agent was able to learn and remain focused on earning a higher cumulative reward value, which represented its ability to achieve goals while minimizing both safety and motion penalty costs.

### 4.5. LLM Prompt for Object Coordinate Extraction

The transition from a natural language query to a metric goal is a two-step process. First, the user’s intent is grounded in a standardized object class via a one-time LLM call. To ensure the output matches the keys stored in the Stage-1 spatial memory, we utilize the specific prompt structure shown in Listing 3.

**Listing 3.** LLM Prompt for Semantic Extraction.

Available detected objects: {bookshelf, fire_hydrant, brick_box}

User question: "{user_query}"


TASK: Extract ONLY the object name.

RULES:

- Return ONLY the exact object_class from the list.

- Match synonyms (e.g., "hydrant" -> "fire_hydrant").

- If no match, return "NONE".



Once the standardized object_class is retrieved, the system shifts to a deterministic rule-based logic. As detailed in the code implementation, the system performs a filtered lookup in the .jsonl database:

1. Filtering: It retrieves all instances matching the extracted class.
2. Proximity Selection: It selects the instance *i* that minimizes the Euclidean distance $d=\sqrt{{\left(x_{i}-x_{r}\right)}^{2}+{\left(y_{i}-y_{r}\right)}^{2}}$ relative to the robot’s current odometry pose $\left(x_{r},y_{r}\right)$.
3. Goal Assignment: The global coordinates $\left[x_{g},y_{g}\right]$ of the selected instance are passed to the TD3 actor as a static target.

### 4.6. VLM Model Characterization and Deployment

In this work, two VLM backbones were evaluated for semantic object localization: GPT-4o mini and Moondream.

- GPT-4o mini (Omni): A closed-source multimodal model developed by OpenAI. GPT-4o mini operates as a cloud-based service accessible via an API, necessitating a persistent high-bandwidth internet connection. While its exact parameter count is proprietary, it is optimized for “omni” performance, processing text and visual data within a single neural network to achieve human-like reasoning. In our framework, the robot acts as a thin client, offloading the heavy computational burden of image analysis to OpenAI’s servers.
- Moondream: Moondream differs from GPT-4o mini as it is small, open-source, and designed for edge deployment. Moondream was developed to run entirely locally on the robot’s onboard computing hardware, meaning that there is no network latency through round trips, and Moondream will continue to operate even when there is no internet coverage available.

**Figure 7:**
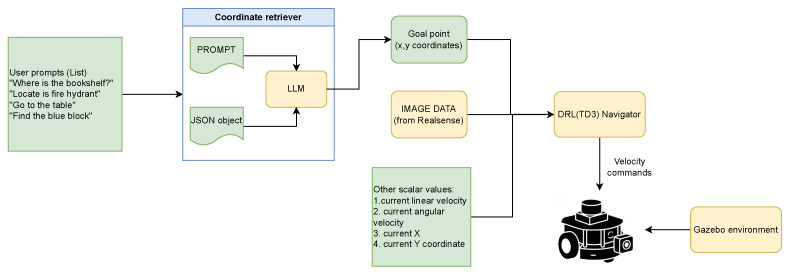
The Stage-2 “Query-to-Action” navigation pipeline. A natural language prompt is resolved by the Coordinate Retriever against the JSON spatial memory to provide global target coordinates. The TD3 DRL agent then utilizes these coordinates alongside real-time depth and odometry data to execute collision-free navigation to the goal.

Here, we introduce their role in the proposed pipeline, while a detailed comparison of their deployment characteristics, information about their number of parameters, and latency is provided in Section 6.2. The second stage of our framework represents the transition from the acquisition of environmental knowledge to the execution of goals. The integrated pipeline (see Figure 7) consists of a Hierarchical Query-Retrieve-Act sequence:

1. Semantic Query Resolution: In this first step, the natural language prompt (e.g., “Where is the fire hydrant?”) is provided to the system. The coordinate retriever parses the query, then makes a Semantic Lookup of the queries on the structured_memory.json database that is created during the exploration phase.
2. Spatial Memory Retrieval: In this second step, the system retrieves the Object from the database based on its Class Label. To make the retrieval process robust, in environments that may contain duplicates of the same type of Object, the retriever uses a priority. The priority is based on the Object’s observation_count (indicating confidence in Identification of Object) and/or the shortest Euclidean Distance from the Robot’s Current Pose.
3. Goal Grounding: Once a match is confirmed, the retrieved global coordinates are extracted and set as the active navigation goal $G=[x_{g},y_{g}]$. These coordinates serve as the target vector for the low-level controller.
4. Closed-Loop DRL Execution: The goal coordinates are fed into the trained TD3 model. In this phase, the agent operates in a closed-loop fashion, continuously fusing the high-level goal vector with real-time 64×64 single-channel depth tensor visual features and 7-dimensional scalar states (as defined in Section 4.1).
5. Dynamic Motion Control: The DRL policy maps these multimodal inputs to continuous velocity commands [v,ω]. While the robot moves toward the retrieved coordinate, the depth-based visual stream ensures the robot avoids unmapped obstacles that were not present during the initial Stage-1 exploration.

This integrated flow bridges the gap between high-level human intent and low-level robotic motion, enabling the Pioneer 3-DX to ground linguistic concepts in physical space. Importantly, Stage 2 does not assume that odometry alone provides absolute global localization: the stored object coordinates are meaningful only when the global reference frame established in Stage 1 is maintained during continuous operation, or re-established via a standard re-localization method (e.g., AMCL/SLAM, visual fiducials, or place recognition). Since wheel odometry is prone to drift in real deployments, it can be fused with an IMU (and other proprioceptive sensors) to improve short-term state estimation. Therefore, the odometry-only configuration in Stage 2 is used to evaluate mapless reactive control once a consistent global frame is available, rather than to claim global localization in a new room. A detailed description of the exact execution procedure for Stage 2, “Query-to-Action” is provided in Algorithm 1.
**Algorithm 1:** Integrated Stage-2 Query-to-Action Pipeline
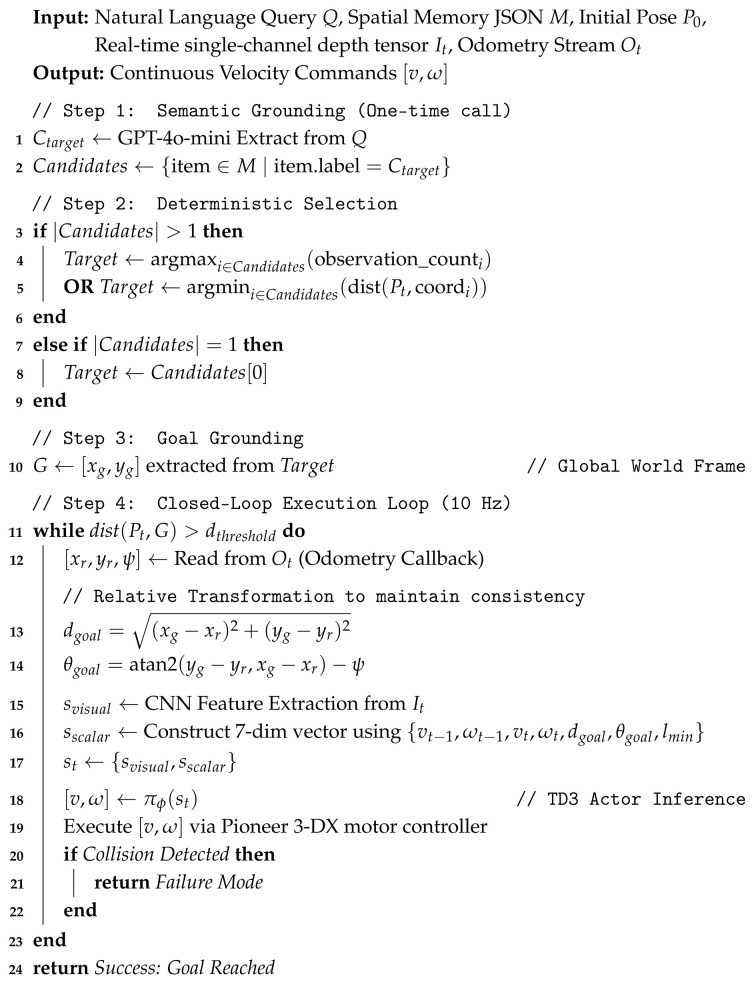


## 5. Testing Phase and Performance Evaluation

In the final phase, we validate the accuracy of the semantic map and the robustness of the DRL-based navigation system based on the success of the robot’s ability to navigate to locations identified during the Stage 1 exploration from random starting positions. The performance of the system is evaluated using four criteria:

(1) SR, which indicates the proportion of trials in which the robot successfully arrived within 1 m of its intended target. We chose 1 m as the threshold for a convenient definition of accessible region rather than a generous coordinate estimation tolerance. As the estimated target coordinate could correspond to the geometric center of an object, this point is not always reachable or safe for the robot to reach, especially for large objects, objects with internal geometries like tables, or objects near walls. Therefore, reaching within 1 m of the target coordinate signifies that the robot can stop at a position near the intended object, which is reachable and free of collisions with the object or other obstacles. It is defined as:

(15) $$SR=\frac{1}{N}\sum_{i=1}^{N}S_{i}$$

where $S_{i}$ is a binary indicator of success for trial *i*.

(2) SPL, which compares the robot’s actual navigation trajectory to an estimated shortest path:

(16) $$SPL=\frac{1}{N}\sum_{i=1}^{N}S_{i}\frac{L_{i}}{\max(P_{i},L_{i})}$$

where $L_{i}$ is the shortest Euclidean distance and $P_{i}$ is the actual Path Length.

(3) Localization Error ($E_{loc}$), which determines the difference in coordinates that the VLM provided for localization as compared with the actual ground truth (Euclidean distance):

(17) $$E_{loc}=\sqrt{{\left(x_{vlm}-x_{gt}\right)}^{2}+{\left(y_{vlm}-y_{gt}\right)}^{2}}$$

where $\left(x_{vlm},y_{vlm}\right)$ are the VLM-derived coordinates and $\left(x_{gt},y_{gt}\right)$ are the ground truth coordinates.

And (4) Finish Time ($T_{f}$), which is the average time taken for the robot to complete the navigation task:

(18) $$T_{f}=\frac{1}{N}\sum_{i=1}^{N}t_{i}$$

where $t_{i}$ represents the time elapsed for each trial *i*.

Table 3 presents a summary of the comparative results of the two VLM backbones in the conducted experiments. Among the approaches compared with Moondream, the former produced the highest reliability with a success rate of 85%, while GPT-4o mini achieved a success rate of only 45%. The success rates of Moondream and GPT-4o mini differed considerably due to their localization error rates; Moondream had a localization error of 0.23 m versus the localization error of 1.75 m for GPT-4o mini. Although GPT-4o mini has a lower success rate, it was very efficient in its successful attempts. In the trials that were successfully completed, GPT-4o mini had an Avg SPL without Fails of 0.70 and a faster average finish time of 13.82 s. This implies that if GPT-4o mini successfully identifies a target, it is likely to move towards it through a shorter path. Therefore, GPT-4o mini’s relatively poor success rates can be attributed to the significant localization error causing the target coordinates to be frequently missed or inappropriately located and, thus, unable to be reached. The data from the experiments indicates that precision in the localization of an agent via a VLM is the most important element of achieving success in ObjectNav.

The observed localization error 0,23 (m) primarily stems from coordinate estimation inaccuracies accumulated during semantic grounding and mapping. This can be mitigated by incorporating a short-range re-localization step that re-triggers VLM-based grounding near the predicted target region, aggregating multi-view observations to refine object coordinates, and applying uncertainty-aware stopping criteria or a final visual servoing stage for precise alignment. It is also important to note that prior studies conducted in highly abstracted simulators such as Habitat report success rates ranging from 21% to 72% [41,42]. In comparison, our ROS-based framework more closely reflects real robotic system constraints, and thus an 85% success rate represents a competitive and meaningful outcome under these more realistic conditions.

Furthermore, the proposed Moondream + DRL framework was compared against a baseline utilizing the Moondream VLM combined with the APF algorithm. As summarized in Table 3, both configurations exhibit an identical localization error of 0.23 m, as they rely on the same semantic grounding backbone. However, the DRL-based approach significantly outperforms the APF baseline in reliability, achieving a success rate of 85% compared with only 42% for the APF method.

The standard deviations in Table 3 were computed over repeated evaluation episodes using the final trained TD3 policy. During evaluation, the robot’s initial pose, target object, and navigation episode conditions were varied, while the trained policy parameters were kept fixed. Therefore, the reported deviations quantify test-time variability caused by different navigation scenarios, semantic localization outputs, and episode-level execution differences. This statistical reporting complements the mean values and provides a clearer indication of the robustness of each VLM-based configuration during evaluation.

Note: For the DRL-based configurations, values are reported as mean ± standard deviation over repeated evaluation episodes using the final trained policy. The APF baseline is reported using mean values only, as it was used as a deterministic planner baseline and was not evaluated under the same repeated-trial statistical protocol.

### 5.1. Trajectory Analysis and Path Visualization

**Figure 8:**
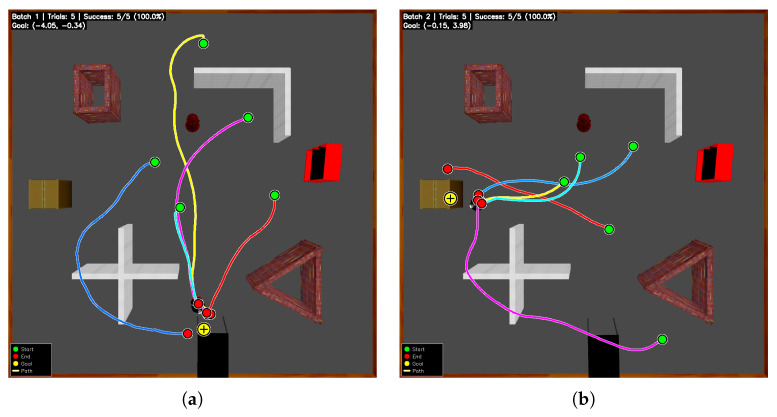
Trajectory results: (**a**) Trials 1–5 showing the table approach; (**b**) Trials 6–10 showing the cardboard box approach. All trials were conducted in the Gazebo simulation environment.

To perform a qualitative evaluation of the DRL agent’s navigation policy, we looked at the paths produced over numerous trials. The multiple colored paths in Figure 8 show that throughout 10 trials with random starting poses, the robot consistently identified and followed a near-optimal direct route to each target while keeping a safe distance between itself and the obstacles. Further, the small amount of variation in the paths produced across the various trials provides evidence that the TD3 policy has developed a consistent relationship between its use of visual and scalar features and its output of velocity commands, which allows for the highest possible SPL while minimizing redundant exploration and oscillatory movements.

### 5.2. Generalization in Unseen Object Environments

**Figure 9:**
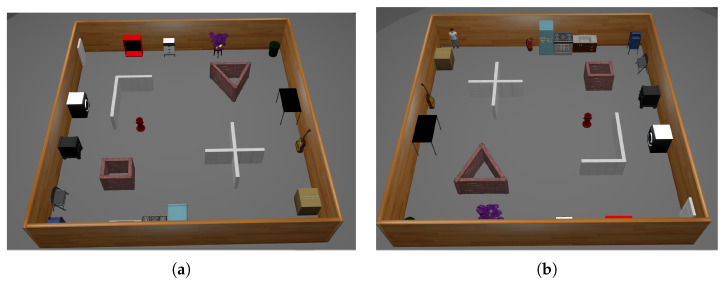
The Gazebo simulation environment configured for multi-object generalization testing and populated with 18 semantically distinct objects: (**a**) top view from one side; (**b**) top view from the opposite side.

In this phase, we evaluate the generalization capabilities of the DRL navigation policy when paired with the Moondream VLM backbone in significantly more complex environments. While the DRL policy was trained on a limited subset of semantic categories in a sparse room, this experiment introduces a comprehensive set of 18 distinct object classes that were not present during the training phase, as shown in Figure 9. The objective is to determine whether the VLM can successfully ground natural language queries into spatial coordinates for a diverse range of objects it recognizes zero-shot, and whether the DRL controller can robustly navigate to these novel geometries amid increased environmental clutter.

The testing environment was augmented with 18 distinct object classes: brown cardboard box, person standing, trash can, extinguisher, fridge, oven, kitchen sink, bookshelf, door, drawer cabinet, fire hydrant, foldable chair, guitar, plant, postbox, safe, table, and washing machine. These objects present diverse scales, shapes, and occupancy signatures, challenging the DRL agent’s obstacle avoidance and goal-reaching behaviors in a densely populated scene. To evaluate navigation performance, 10 objects were randomly selected from the full set of 18, and the reported metrics are averaged over navigation trials conducted across these 10 target objects.

**Figure 10:**
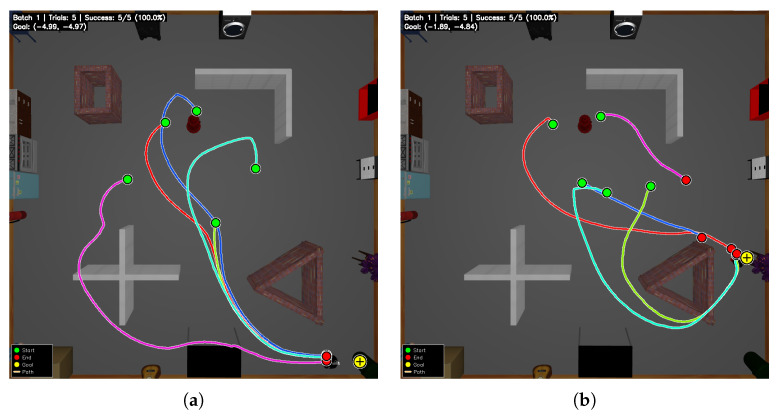
Trajectory results in the 18-object environment: (**a**) navigation trials targeting the trash can; (**b**) navigation trials targeting the plant. All trials demonstrate the agent’s ability to generalize to dense, cluttered scenes with novel semantic targets.

As shown in Table 4 and visualized in Figure 10, the Moondream-based architecture achieved an overall success rate of 0.68, with an average SPL of 0.47 (including failed episodes) and 0.74 (successful episodes only). Compared with the earlier sparse-object evaluation, the reduced success rate reflects the increased navigational difficulty posed by dense clutter and narrow passages between objects. Nevertheless, the high SPL among successful trials indicates that when the agent reaches its target, it does so via near-optimal paths.

Table 5 presents the per-object success rates for the 10 randomly selected target objects. The results reveal considerable variation across object categories, with fridge and guitar achieving the highest success rates of 0.9, while extinguisher and safe proved most challenging at 0.5. This variation can be attributed to differences in object size, visual distinctiveness for VLM-based grounding, and the surrounding obstacle geometry affecting navigability.

### 5.3. Obstacle Stress Test

To further assess the robustness of the learned TD3 navigation policy under adverse conditions, we conducted an obstacle stress test in which the environment was densely populated with additional static obstacles beyond the semantic target objects. This experiment evaluates whether the depth-based DRL controller can maintain reliable collision avoidance and goal-reaching behavior when the free navigable space is substantially reduced by cluttered obstacle placements.

**Figure 11:**
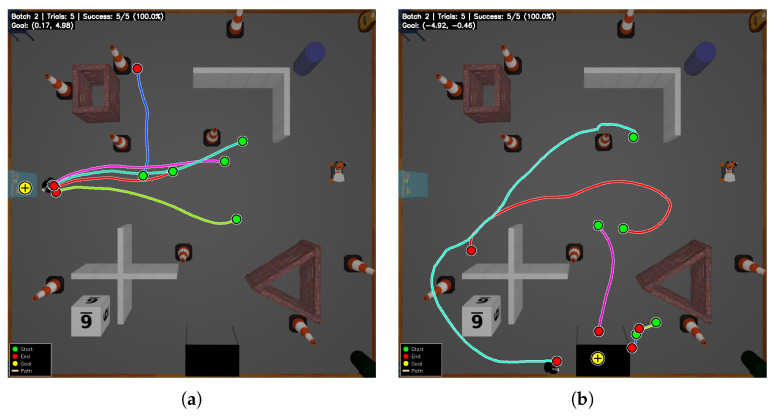
Trajectory results under obstacle stress conditions: (**a**) navigation trials targeting the fridge area amid dense obstacle placement; (**b**) navigation trials targeting the table with additional clutter. The agent demonstrates reactive obstacle avoidance while maintaining goal-directed behavior.

As summarized in Table 6 and illustrated in Figure 11, the system achieved a success rate of 0.68, with an average SPL of 0.56 (including failures) and 0.88 (successful episodes only). These results are consistent with the multi-object generalization experiment, suggesting that the primary challenge arises from navigating in confined spaces rather than from semantic grounding errors. The high SPL among successful trials confirms that the TD3 policy retains its path-efficiency even under significantly increased obstacle density, leveraging its depth-based visual stream for reactive collision avoidance in cluttered corridors and narrow passages.

### 5.4. Sign-Based Semantic Navigation

In addition to navigating toward physical objects, we evaluated the system’s ability to navigate toward semantic signs-visual indicators placed within the environment. Five sign-type objects were introduced: parking sign, stop sign, speed limit sign, toilet sign, and exit sign. Unlike physical objects with well-defined 3D geometries, signs are flat, wall-mounted or pole-mounted visual markers that present distinct challenges for both VLM-based detection (due to their planar nature and smaller visual footprint) and depth-based coordinate estimation. This experiment tests whether the VLM can reliably detect and localize sign-type targets, and whether the DRL controller can navigate to their estimated positions.

**Figure 12:**
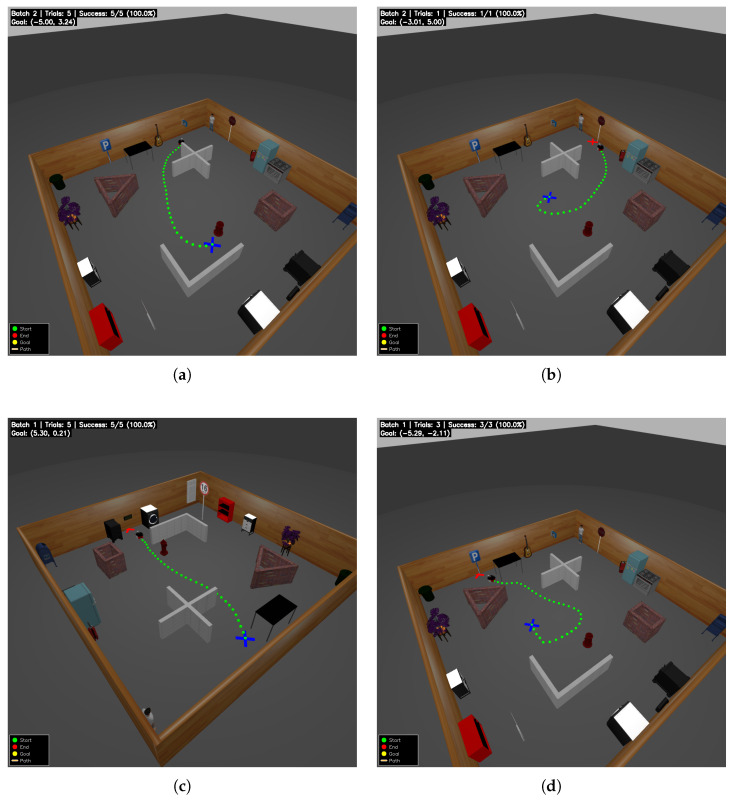
Trajectory results for sign-based navigation: (**a**) WC (toilet) sign; (**b**) stop sign; (**c**) exit sign; (**d**) parking sign. The dotted green paths indicate successful goal-reaching trajectories in an environment populated with diverse objects and sign-type targets.

As shown in Table 7 and visualized in Figure 12, the sign-based navigation experiments yielded a success rate of 0.66, with an average SPL of 0.51 (including failures) and 0.77 (successful episodes only). Notably, the sign navigation results slightly outperform the multi-object and obstacle stress experiments in both success rate and path efficiency. This suggests that the VLM is capable of grounding non-object semantic concepts—such as directional or informational signs including parking, stop, speed limit, toilet, and exit indicators—into spatial coordinates with sufficient accuracy for downstream DRL-based navigation. The higher SPL among successful trials further indicates that sign targets, often positioned near walls or in corridor junctions, provide relatively unobstructed approach paths once correctly localized.

### 5.5. Generalization in Unseen Environment Layout

**Figure 13:**
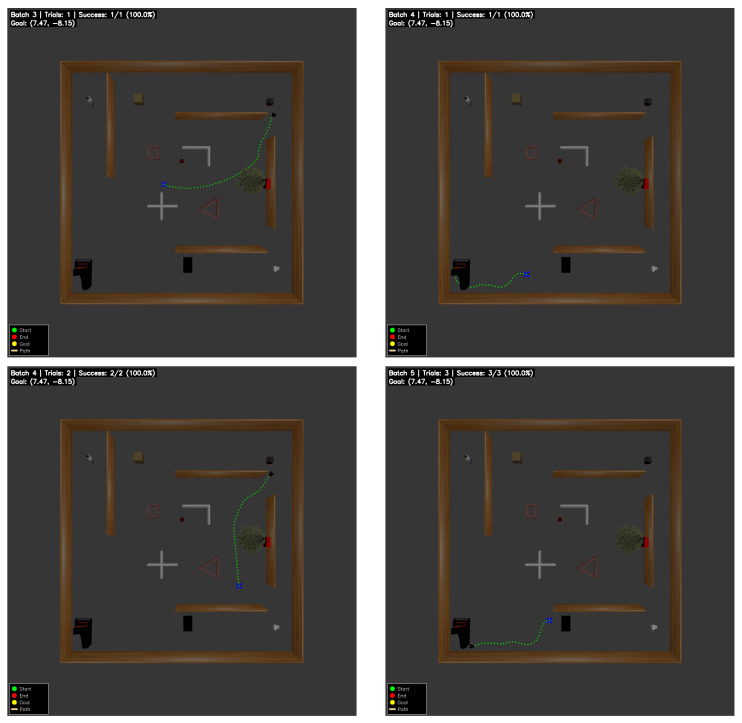
The multi-room generalization environments. The layouts include multiple rooms and narrow corridors to test the agent’s ability to navigate unmapped, complex topographies over 100 episodes.

The generalization performance of the TD3 policy was tested rigorously using a new and more complex unseen environment. Unlike the original training environment, which was a single 5 m × 5 m room, the generalization environment contains a larger enclosed layout with internal wall segments, narrow passages, and furniture-like obstacles, as shown in Figure 13. Its internal partitions and obstacle distribution create a more challenging navigation scenario than the basic training room. The increased layout complexity requires the TD3 policy to perform more robust obstacle avoidance and goal-directed navigation in previously unseen spatial configurations.

Twenty-five independent test episodes were created to evaluate how the TD3 agent would operate in this new complex environment. During each test episode, both the robot and the target object were initialized at random locations within the new environment, requiring the robot to perform significantly more challenging long-range navigation. Unlike the original training environment, where the maximum navigation area was limited to a 5 m × 5 m space, the policy was evaluated in a substantially larger 15 m × 15 m environment, resulting in longer travel distances and increased navigation complexity. For the purpose of determining how well the TD3 agent performed, two types of performance metrics were used: SR and SPL over the course of an episode.

As shown in Table 8, the transition from the original 5 m × 5 m single-room training environment to the more complex unseen environment challenged the agent, with the SR decreasing from 0.79 to 0.6. This reduction reflects the increased difficulty caused by internal wall segments, narrow passages, and unseen obstacle arrangements. At the same time, the Avg. SPL value of 0.86 without failed episodes indicates that, when the agent successfully reached the target, it was generally able to follow efficient trajectories.

The transition from the original training room to the complex unseen environment was therefore challenging for the TD3 agent, mainly due to the increased spatial complexity and longer navigation distances required to reach the target. Compared with the original 5 m × 5 m single-room training environment, the unseen layout required the agent to follow longer trajectories through a less familiar spatial configuration while maintaining goal-directed navigation. These results, shown in Table 8, indicate that the DRL-based policy was still able to handle extended navigation distances, although the increased spatial complexity made the task more difficult.

It should be noted that the generalization experiment evaluates only the DRL-based navigation policy under unseen room configurations, without re-estimating object coordinates via the VLM. Although performance decreases to SR (0.6) in the complex unseen layout due to the increased spatial complexity and longer required navigation paths, the agent still retained meaningful goal-directed navigation capability. Prior studies [43] on similar robotic platforms have demonstrated that DRL policies trained under comparable conditions can successfully transfer to modified environments and maintain goal-directed navigation, including avoidance of unseen and dynamic obstacles. The complete operation of the proposed framework, including semantic object localization, coordinate storage, and TD3-based navigation, is demonstrated in Supplementary Video S1. The corresponding implementation files, source code, and configuration scripts are available in Supplementary Source Code S1.

## 6. Analysis and Comparison

### 6.1. Qualitative Analysis of VLM Localization Errors

To understand the root causes of the substantial localization error gap between GPT-4o mini (1.75 m) and Moondream (0.23 m), we conducted a detailed qualitative analysis of the VLM outputs across multiple detection instances. The analysis reveals that the high localization error in GPT-4o mini stems primarily from limitations within the VLM’s internal visual grounding layer rather than from downstream processing in the coordinate estimator. Specifically, we identified four recurring failure modes in GPT-4o mini’s object localization:

(1) Inaccurate pixel-center estimation: GPT-4o mini frequently miscalculates the geometric centers of correctly identified objects. Even when the correct object class is returned, the reported pixel coordinates (u,v) are displaced from the true object center, sometimes by over 100 pixels. Since the coordinate estimator samples depth values from a 5×5 neighborhood around the reported pixel center, an offset pixel location retrieves depth information from a different surface or background region, directly inflating the world-coordinate error.

(2) Object misclassification: In several instances, GPT-4o mini completely misidentifies neighboring objects. For example, a wooden chair may be labeled as a “table”, or an exit sign may be classified as a “fire hydrant”. When the system records coordinates for a misclassified object, the resulting world position corresponds to an entirely different physical entity, producing localization errors that can exceed 2 m.

(3) Depth ambiguity from occluding objects: When a target object is partially occluded by a foreground obstacle (e.g., a table visible behind a wooden chair), GPT-4o mini tends to estimate the pixel center at a location influenced by the occluder. The depth camera then returns the distance to the foreground object rather than the intended target, resulting in an incorrect world coordinate that reflects the occluder’s position.

(4) Spurious detections of non-target objects: GPT-4o mini occasionally detects objects not present in the target list (e.g., labeling a wall-mounted sign as a “safe”), and the reported coordinates for these phantom detections further degrade the average localization accuracy when they are erroneously associated with target categories.

In contrast, Moondream demonstrated significantly more precise pixel-center estimation for the same objects, despite its smaller parameter count (1.86 B vs. ≈8 B). We attribute this to Moondream’s SigLIP-based vision encoder, which was specifically designed for grounded visual understanding tasks, whereas GPT-4o mini’s general-purpose architecture appears to prioritize high-level semantic reasoning over fine-grained spatial localization. Importantly, both VLMs use the identical downstream coordinate estimation pipeline (depth extraction, frame transformation, and temporal filtering), confirming that the performance difference originates in the VLMs’ visual grounding output rather than in the geometric processing.

### 6.2. Computational Cost and Inference Latency Analysis

To characterize the real-time performance of each VLM backbone, we recorded per-frame inference latency over 100 consecutive stop-and-sense cycles during the exploration phase. The latency encompasses the full round-trip time from image submission to coordinate output receipt, including network transmission overhead for GPT-4o mini and local GPU inference time for Moondream. Table 9 summarizes the results.

While GPT-4o mini delivers a marginally lower median latency (0.86 s vs. Moondream’s 1.05 s) due to optimized cloud infrastructure, it exhibits substantially higher variance (σ=0.47 s) and is vulnerable to severe latency spikes, reaching a maximum of 4.80 s. These outliers are attributable to network congestion, API rate limiting, and variable server load—factors inherently beyond the robot’s control. In contrast, Moondream offers exceptional predictability, with a maximum latency capped at 1.51 s and a standard deviation of only 0.27 s, making it significantly more suitable for real-time robotic applications where consistent inference timing is critical for maintaining the stop-and-sense cycle cadence.

From a deployment perspective, GPT-4o mini requires continuous internet connectivity and incurs per-query API costs, whereas Moondream runs entirely on-device (approximately 5 GB VRAM at FP16 precision) with zero network dependency. For indoor service robotics applications with limited onboard resources or unreliable network connectivity, Moondream is the more suitable VLM backbone because it provides lower localization error, a higher navigation success rate, and more predictable inference latency. Table 10 presents the extended comparison.

### 6.3. Discussion: Comparison with State-of-the-Art Semantic ObjectNav Methods

A direct quantitative comparison with state-of-the-art semantic ObjectNav methods is limited by substantial differences in evaluation platforms, sensing assumptions, robot embodiment, and benchmark protocols. Recent zero-shot and language-guided ObjectNav approaches [18,19,20,21,22,25,30] are commonly evaluated in photo-realistic embodied-AI simulators such as Habitat [44], using standardized datasets such as HM3D, Gibson, and MP3D. These environments are well suited for controlled benchmark comparison, but they generally abstract away several system-level factors that are important for robotic deployment, including ROS communication, sensor-actuator synchronization, robot kinematics, real-time control loops, odometry handling, and coordinate-frame transformations.

To provide a fairer comparison despite these differences, we added a qualitative/proxy comparison with representative ObjectNav and VLM-based navigation methods, as summarized in Table 11. Instead of directly comparing success rates across incompatible platforms, the table compares the methods in terms of evaluation environment, robot/system realism, semantic grounding, memory representation, and navigation execution. This highlights the main distinction of the proposed work: while prior methods primarily target benchmark performance in Habitat/Gibson-style environments, our framework is implemented and evaluated in a ROS/Gazebo-based robotic simulation with a physically modeled Pioneer 3-DX platform, RGB-D sensing, 2D LiDAR, odometry, and differential-drive motion constraints.

In this context, the contribution of our work is not intended to replace standardized ObjectNav benchmarking, but to demonstrate a deployment-oriented semantic navigation pipeline that integrates VLM-based object coordinate estimation, LiDAR-assisted semantic memory construction, and TD3-based goal-directed navigation within a ROS-compatible robotic system. To the best of our knowledge, prior VLM-based semantic ObjectNav studies have not demonstrated this complete combination in a ROS/Gazebo environment with a physically simulated mobile robot.

While the absolute success rates are not directly comparable across platforms, the architectural contributions of our work—decoupled VLM-based semantic grounding, persistent object-level memory, and map-free TD3 navigation—address the same core challenges identified in Habitat-based literature (perception noise sensitivity, runtime VLM inference cost, long-horizon planning), but through a deployment-oriented design that prioritizes real-time feasibility and system-level integration over benchmark-optimized performance.

### 6.4. Object Recognition Module Comparison

We also compared the VLM-based recognition module with suitable open-vocabulary computer vision object detection methods, namely Grounding DINO and YOLO-World v2.6. These methods were tested for recognizing surrounding objects in the simulated environment. However, in several cases, they produced incorrect labels or inaccurate image-level detections, which made reliable estimation of the target object position difficult.

**Figure 14:**
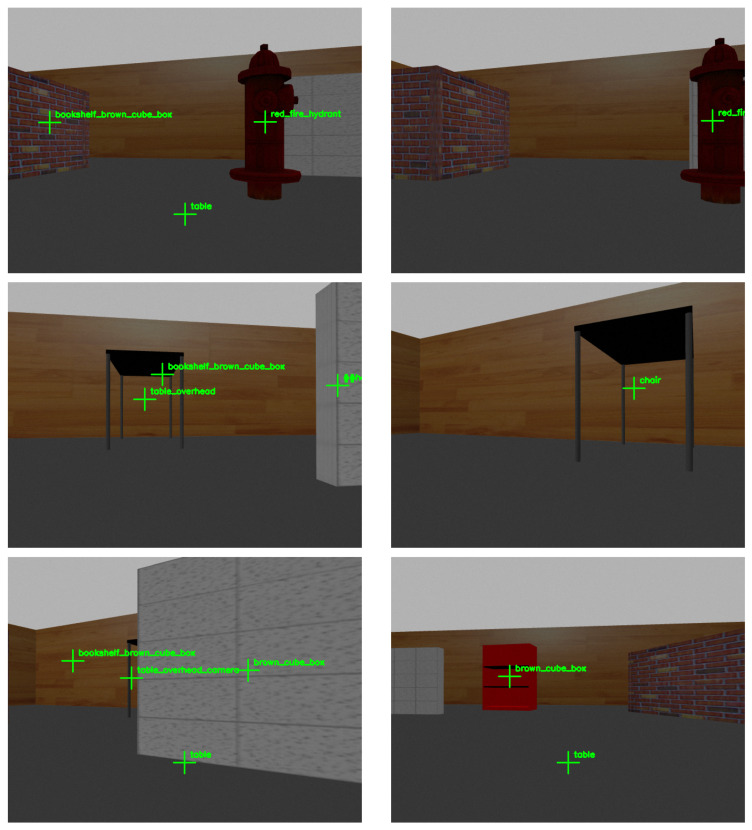
Qualitative object-recognition results obtained using YOLO-World and Grounding DINO.

As shown in Figure 14, the second image in the first row and the second image in the second row correspond to YOLO-World v2.6 results. In the first case, the model failed to correctly recognize the target object, where it says chair instead of table; in the second image, it could correctly recognize. Moreover, we did other tests but did not provide labels at all, and therefore the results were not included in the quantitative object-localization comparison. The remaining examples correspond to Grounding DINO, where the model was able to detect some objects but also produced multiple incorrect detections, despite several correct labels being present.

It should also be noted that these detector-based methods are primarily designed for object detection from structured text prompts or class names. They do not directly provide the same level of natural language grounding as a VLM, especially for more flexible human instructions or potential voice-to-text commands. In contrast, the proposed VLM-based framework can interpret natural language commands and convert them into structured semantic target information for the DRL navigation policy. This makes the proposed approach more suitable for human-oriented semantic navigation, where the input command may be less structured than a conventional object-detection prompt.

### 6.5. Component Contribution Analysis

Although a full ablation study systematically removing each component was not conducted in this work, the experimental results provide insight into the relative contribution of each architectural component. The comparison between GPT-4o mini and Moondream (Table 4) effectively ablates the VLM backbone while holding all other components constant (identical coordinate estimator, TD3 policy, and sensor configuration), demonstrating that VLM localization accuracy is the dominant factor in overall system success (a 3.3× reduction in localization error yields a 3.8× improvement in success rate).

The depth-only visual input design (single-channel 64×64 depth image rather than RGB) was motivated by the need for appearance-invariant generalization, and the consistent navigation performance supports this design choice. A comprehensive ablation study examining individual reward components, observation modalities, and temporal filtering parameters is planned for future work.

## 7. Discussion, Limitations and Future Work

The primary objective of this work was to demonstrate that object coordinate estimation can be achieved by integrating odometry, 2D LiDAR, and VLM-based semantic grounding, and that the memorized object locations can then serve as navigation targets using the same RealSense camera and odometry sensors within a unified framework. While GPT-4o mini showed strong performance, Moondream, a significantly smaller 1.8B-parameter model, also achieved competitive results, indicating the feasibility of lightweight VLMs for embodied navigation.

Several limitations remain. In the current experiments, all target objects were static during both exploration and navigation. In practical environments, objects may move after exploration, making stored semantic coordinates outdated. To address this, the framework can be extended with closed-loop semantic verification. When the robot reaches a verification radius around the stored target coordinate, the VLM can be triggered again to confirm whether the target is visible and consistent with the user command. If confirmed, the task is completed. If the target is missing, occluded, or detected elsewhere, the VLM re-estimates the coordinate from the current viewpoint, updates the semantic memory, and provides the new goal to the TD3 policy. Since TD3 uses goal-relative observations, this online coordinate update does not require policy retraining.

Although the framework was evaluated through enlarged-room generalization, obstacle stress testing, object-level stress testing, and sign-recognition-based navigation, all experiments were conducted in static simulation environments. Therefore, the main remaining limitation is the absence of evaluation in dynamic indoor scenarios with moving humans, moving obstacles, or continuously changing object locations. Nevertheless, prior work [43] demonstrated that a DRL navigation policy trained under comparable conditions, with a similar robotic platform and sensor modalities, can transfer to a real robot and reactively adapt to unseen and dynamic obstacles. Future work will therefore extend the proposed VLM-guided DRL framework to dynamic real-world environments where semantic perception and navigation must remain robust under changing spatial conditions. In addition, although the fixed 1.0 m arrival threshold provides a practical accessible-region criterion for large, wall-adjacent, or partially inaccessible objects, future work may introduce an adaptive object-specific threshold based on object geometry, physical size, placement, and safe reachable distance.

Simulation enabled controlled and repeatable evaluation of object coordinate estimation, goal-directed navigation, layout generalization, obstacle and object stress testing, and sign-based navigation. However, it does not fully reproduce real-world sensing conditions. RGB-D sensors such as Intel RealSense may produce noisy depth, incomplete object boundaries, missing values, and blind spots, especially near object edges, reflective surfaces, or low-texture regions. These artifacts can affect object localization and introduce uncertainty into navigation.

These issues are expected engineering challenges rather than fundamental limitations of the framework. On a physical robot, they can be mitigated through depth filtering, temporal averaging, outlier rejection, sensor calibration, obstacle safety margins, and confidence-based validation of estimated object coordinates. Thus, although real-world deployment was outside the current scope, the framework remains compatible with practical robotic implementation.

In real-world navigation, odometry alone is insufficient because of cumulative drift. This can be reduced by fusing wheel odometry with inertial measurements using an Extended Kalman Filter. For larger indoor environments with multiple possible routes, a global path-planning layer can also be added to generate intermediate waypoints or a coarse route toward the estimated object location. In this hierarchical architecture, the global planner handles long-range route selection, while the DRL policy acts as a local reactive controller for short-range goal following and obstacle avoidance. This is important because the current TD3 policy is not designed for global path planning in large-scale environments with multiple alternative paths. With a physical robot, remaining sensing, localization, and long-range navigation challenges can therefore be addressed through sensor fusion, filtering, calibration, global planning, and real-world fine-tuning.

The current system also relies on direct VLM-based coordinate estimation without long-term structured memory. Although this demonstrates perception-to-navigation feasibility, performance may degrade as the number of objects or environment size increases. Future work will explore retrieval-augmented generation mechanisms with structured JSON-like object memories to store, retrieve, and update object coordinates more efficiently over time. Sequential commands, such as navigating to object A, completing an action, and then moving to object B, will also be investigated.

Finally, although a policy trained in a single room generalized to enlarged-room navigation, the diversity of indoor conditions remained limited. Future work will expose the navigation policy to more varied layouts, lighting conditions, and object arrangements to improve robustness and generalization in real-world environments.

## 8. Conclusions

In this work, we presented a mobile robot navigation framework that integrates odometry, a 2D LiDAR, an Intel RealSense depth camera, and a locally deployed VLM for object coordinate estimation, demonstrating the feasibility of fully local perception and reasoning. In addition, we trained a custom TD3-based navigation policy with a task-specific reward function that enables goal-directed navigation using only odometry and RealSense depth images, without reliance on global maps during execution.

Through extensive evaluation, we observed that the Moondream VLM achieves better coordinate estimation performance with fewer parameters than GPT-4o-mini, making it well suited for local deployment on resource-constrained robotic platforms. Specifically, Moondream reduced target object localization error by 7.6× times compared with GPT-4o-mini, which in turn led to a 1.8× higher navigation success rate due to more accurate goal coordinate estimation, with success rates of 0.85 for Moondream + DRL, 0.45 for GPT-4o-mini + DRL, and 0.42 for Moondream + APF. In the 2nd stage, the target-coordinate-driven TD3 navigation and depth-based mapless navigation were well aligned with the proposed VLM-based object detection and coordinate estimation pipeline, demonstrating coherent integration between perception and control.

While many existing works employ vision transformers or VLMs accessed through cloud APIs and evaluate their methods in highly abstracted simulation environments, zero-shot navigation in such approaches typically relies on standard global and local path planners operating over complex multi-layer 2D or 3D semantic maps. In contrast, our approach uses a 2D map only during an initial exploration phase with a conventional planner, followed by fully mapless TD3-based navigation in the second stage, which is semantically guided by VLM-derived object coordinates estimated during the first stage. To the best of our knowledge, such work was first introduced in our work with a locally running VLM with coordinate estimation and navigation with a vision-based TD3 policy.

The proposed framework has the potential to significantly impact indoor navigation scenarios, particularly those involving voice-based or natural language commands, where a robot must simultaneously perceive, understand, and reason about its environment based solely on onboard sensing and locally executed models.

## Figures and Tables

**Table 1:** Schema of the Semantic Object Database.

Field	Description
id	Unique identifier (e.g., obj_000000)
object_class	Semantic label from VLM (e.g., “fire hydrant”)
confidence	Detection score (0.0–1.0)
world_x, y, z	Global 3D coordinates in the map frame
pixel_x, y	Center pixel coordinates in the source image
robot_x, y, θ	Robot’s pose at the moment of capture
observation_count	Number of times this object was confirmed

**Table 2:** TD3 Training Hyperparameters.

Parameter	Value
Total Timesteps	200,000
Optimizer	Adam
Learning Rate	$1\times 10^{-4}$
Batch Size	256
Replay Buffer Size	1,000,000
Action Noise (σ)	0.8
Discount Factor (γ)	0.99
Polyakh Averaging (τ)	0.005

**Table 3:** Comparative performance of VLM backbones across navigation metrics. Values are reported as mean ± standard deviation, where standard deviation was computed over repeated evaluation trials using the final trained policy.

Model	Avg SPL	Avg SPL	Success Rate	Loc. Error	Finish Time
	All Trials	Success Only			
GPT-4o mini + DRL	0.32±0.38	0.70±0.21	0.45±0.50	1.75±1.57 m	13.82±11.12 s
Moondream + DRL	0.64±0.32	0.75±0.19	0.85±0.36	0.23±0.08 m	22.37±14.86 s
Moondream + APF	0.34	0.82	0.42	0.23 m	25.98 s

**Table 4:** Navigation Performance in the 18-Object Generalization Environment.

Model	Avg SPL	Avg SPL (Success Only)	Success Rate
Moondream + DRL	0.47	0.74	0.68

**Table 5:** Per-Object Success Rates for the 10 Randomly Selected Target Objects.

Object Name	Success Rate
Fridge	0.9
Guitar	0.9
Bookshelf	0.7
Door	0.7
Table	0.7
Trash Can	0.7
Plant	0.6
Washing Machine	0.6
Extinguisher	0.5
Safe	0.5
Average	0.68

**Table 6:** Navigation Performance under Obstacle Stress Conditions.

Model	Avg SPL	Avg SPL (Success Only)	Success Rate
Moondream + DRL	0.56	0.88	0.68

**Table 7:** Navigation Performance for Sign-Based Semantic Targets (evaluated across 5 sign objects: parking sign, stop sign, speed limit sign, toilet sign, and exit sign).

Model	Avg SPL	Avg SPL (Success Only)	Success Rate
Moondream + DRL	0.51	0.77	0.66

**Table 8:** Generalization Performance across 25 Episodes in an enlarged unseen Environment.

Metric	Training Env	Generalization in Enlarged Room
Success Rate (SR)	0.85	0.6
Avg. SPL	0.75	0.86 (Without fails)

**Table 9:** Inference Latency Statistics of VLM Backbones (100 cycles).

Model	Mean (s)	Median (s)	Std Dev (s)	Min (s)	Max (s)
GPT-4o mini	0.97	0.86	0.47	0.59	4.80
Moondream	1.03	1.05	0.27	0.47	1.51

**Table 10:** Extended Comparison of VLM Backbones across All Metrics.

Metric	GPT-4o Mini	Moondream
Success Rate	0.45	0.85
Avg SPL	0.63	0.53
Localization Error (m)	1.75	0.23
Avg Finish Time (s)	16.6	19.13
Median Inference Latency (s)	0.86	1.05
Max Inference Latency (s)	4.80	1.51
Latency Std Dev (s)	0.47	0.27
Parameters	≈8 B	≈1.86 B
Execution Mode	Cloud (API)	Local (On-device)
Internet Required	Yes	No

**Table 11:** Qualitative/proxy comparison with representative ObjectNav and VLM-based semantic navigation methods evaluated under different benchmark settings, including Habitat/Gibson/HM3D/MP3D and ROS/Gazebo physical robot simulation.

Method/Category	Evaluation Environment	Embodiment and System Realism	Semantic Grounding	Memory and Map Representation	Navigation Policy/Planner
Habitat-based ObjectNav benchmark methods [18,19,20,21,22,44]	Habitat with HM3D, Gibson, or MP3D-style datasets.	Photo-realistic embodied-AI simulation with benchmark-defined sensing and actuation.	Object-category or language-conditioned target specification.	Learned maps, topological memory, semantic memory, or simulator-level scene priors, depending on the method.	Global planning, learned policy, or hybrid planner in benchmark simulation.
VLM/LLM-based zero-shot ObjectNav methods [25,30]	Primarily Habitat/HM3D/MP3D-style evaluation environments.	Embodied-AI simulation focused on semantic reasoning and visual–language grounding, generally without ROS-level robotic middleware.	VLM/LLM-based open-vocabulary target interpretation.	High-level semantic memory, frontier-based exploration, topological representation, or planner-based reasoning.	Planner-based or hybrid semantic navigation pipelines, typically evaluated under abstracted simulator assumptions.
Proposed Moondream + DRL framework	ROS/Gazebo realistic robotic simulation.	Physically simulated Pioneer 3-DX robot with RGB-D sensing, 2D LiDAR, odometry, differential-drive kinematics, and ROS communication.	Edge-deployable VLM-based object recognition and coordinate estimation from visual observations.	Persistent JSON semantic coordinate memory constructed during exploration using VLM-based object labels and estimated world coordinates.	TD3-based goal-directed mapless navigation during execution using compact goal-relative observations and onboard sensing.

## Data Availability

No new data were created or analyzed in this study.

## Supplementary Materials

The following supporting information can be downloaded at: Video S1: Demonstration of the proposed VLM-guided semantic navigation framework in the Gazebo simulation environment, available at https://youtu.be/LQ-CnGsYuNE (accessed on 20 May 2026); Source Code S1: Source code, implementation files, and configuration scripts for the proposed framework, available at https://anonymous.4open.science/r/Yernar-SDU-VLM_project-VLM_DRL-based-autonomous-robot-ObjectNav-4270/README.md (accessed on 20 May 2026).
